# New insights into photoactivated volume generation boost surface morphing in liquid crystal coatings

**DOI:** 10.1038/ncomms9334

**Published:** 2015-09-21

**Authors:** Danqing Liu, Dirk J. Broer

**Affiliations:** 1 Institute for Complex Molecular Systems (ICMS), Eindhoven University of Technology, Den Dolech 2, 5612 AZ Eindhoven, The Netherlands; 2 Laboratory of Functional Organic Materials and Devices (SFD), Department of Chemical Engineering and Chemistry, Eindhoven University of Technology, Den Dolech 2, 5612 AZ Eindhoven, The Netherlands

## Abstract

Photoactivated generation of disorder in a liquid crystal network produces free volume that leads to the controlled formation of dynamic corrugations at its surface. The liquid crystal order amplifies the deformation of copolymerized azobenzene, which takes place on molecular length scales, to a micrometre-sized macroscopic phenomenon based on changes in density. We postulate a new mechanism in which continuous oscillating dynamics of the *trans*-to-*cis* isomerization of the azobenzene overrules the net conversion, which is currently considered as the origin. This is supported by a significant local density decrease when both the *trans* and *cis* isomers are triggered simultaneously, either by dual-wavelength excitation or by the addition of a fluorescent agent converting part of the light to the *cis*-actuating wavelengths. This new insight provides a general guideline to boost free volume generation leading not only to larger macroscopic deformations but also to controllable and faster non-equilibrium dynamics.

## Abstract (web summary)

The photomechanical surface modulation of liquid crystal polymer networks can be amplified with a small amount of azobenzene, but its mechanism is not yet fully understood. Here, Liu and Broer propose that the continuously oscillating *trans*-to-*cis* isomerization of azobenzene plays the dominating role.

## Graphical abstract

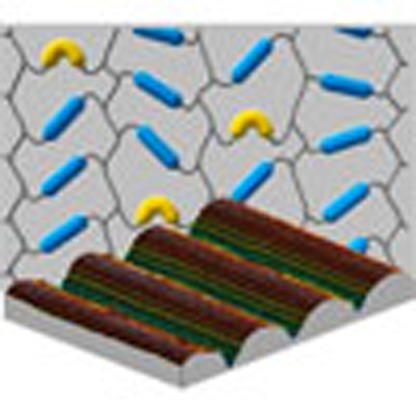

In materials science, the amplification of collective molecular motions from the nanoscopic to the macroscopic scale is one of the most riveting challenges not only leading to a better understanding of biological mechanisms but also to new functionalities in, among others, robotics and haptics. Research on molecular machines has mainly focused on the movement of individual molecules with the emphasis to amplify the effects obtained at the single molecular level. In contrast, stimuli responsive materials are starting at the macroscopic level. By adequate modification, the materials will adapt their shape by external stimuli. In responsive liquid crystal polymer networks, the individual molecular motion is amplified by molecular cooperativity to macroscopic level.

Azobenzene is among the most studied photomechanical molecules. It is characterized by a reversible transformation between its *trans* and *cis* isomers. When illuminated with ultraviolet light, preferably in its maximum absorption band at 365 nm, it undergoes a transition from its ground *trans* state to the less stable *cis* state. The reversed reaction from the *cis* to the *trans* state can occur thermally or by illumination with visible light in the absorption band of *cis* isomer at around 455 nm. The isomerization of azobenzene proceeds with a geometrical change on molecular level. From *trans* (elongated) to *cis* (bent) state, the molecular length changes from 9 to 5.5 Å ref. 1. This on itself might result in relatively small geometric changes as observed in isotropic polymers containing azobenzene crosslinkers^2^,^3^. Earlier work has shown the possibility to amplify the conformational change of azobenzene molecules to the macroscopic level by integrating it into a liquid crystal elastomer^4^ or a glassy liquid crystal network (LCN)^5^. Even small azobenzene concentrations as low as 2 w% lead to significant deformations of these polymer networks illustrating the amplification effects of the ordered network^6^. Many other morphing behaviours (bending or curling) were demonstrated by a number of groups^7^,^8^,^9^,^10^,^11^,^12^.

The current mechanism for the photomechanical effects of azobenzene-modified liquid crystal polymers is based on the reduction of the order parameter when the *cis* isomer is formed^13^. Nematic liquid crystals tend to orient towards a common direction (director). The *trans* azobenzene isomer adapts the alignment of the LCN. While illuminated with ultraviolet light, the *cis* isomer with its bent conformation reduces the degree of order, as quantified by order parameter, and builds up stresses leading to an anisotropic dimensional change of the LCN with a contraction along the average molecular orientation and expansion to the two perpendicular directions^14^, eventually accompanied with some out of plane deformation of the azobenzene moieties as observed in azobenzene side-chain polymers. 15

Although these mechanisms quite well explain the deformation of free-standing polymer liquid crystal films, some observed phenomena still remain veiled. For instance, the local deformation of azobenzene-modified coatings by light exposure^16^ cannot be explained by changes of the order parameter alone. Also, a large mismatch in response time is found between the azobenzene chemistry and the corresponding mechanical deformation. In this paper, we address these issues by introducing the dynamics of the *trans–cis* oscillation of the azobenzene in relation to free volume effects. Considering these new insights, we developed novel technologies to enhance on the effect of mechanical deformation.

## Results

### Kinetic mismatch azobenzene chemistry/surface deformation

**Figure 1:**
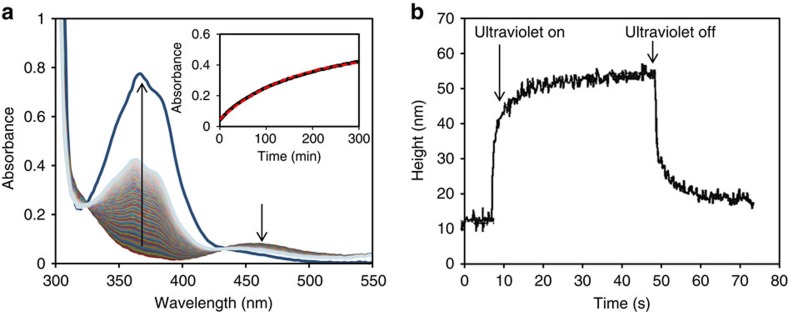
Kinetic mismatch between photochemistry and mechanical response. (**a**) Change in absorbance during thermal relaxation in dark of the *cis* to the *trans* state of azobenzene in an LCN* measured at room temperature. The insert shows the change in absorption with time fitted by a single exponential decay of the *cis* state. (**b**) Mechanical response measured as a height change of the thin film versus time upon actuation by 365 nm light of intensity 78 mW cm^−2^ and a subsequent relaxation in dark. The film thickness is 4 μm, the corresponding density decrease is 1.3%.

Recently, we published that the order parameter reduction goes along with a density decrease/volume increase^16^. In this study, we actuated a chiral-nematic (cholesteric) LCN further denoted as LCN*. In an LCN*, the molecular rod-like moieties rotate along a helix axis perpendicular to the film surface that provides an in-plane symmetry. When subjected to the light-induced loss of order, the volume change results solely from expansion along the helix axis, as the in-plane deformation is zero^17^. The density change was measured by ultraviolet exposing azobenzene containing LCN* films immersed in salt brine. During illumination, polymer film starts floating, indicating the density decrease. As soon as the light source is switched off, the film sinks again within 10 s. According to current theory, this fast restoration (in seconds) to the original high density should imply a fast *cis*-to-*trans* back isomerization process (relaxation). However, when we record the *cis*-to-*trans* relaxation in the LCN* by taking ultraviolet–visible spectra(Fig. 1a; *vide infra*), we find that relaxation is in the order of hours. The relaxation can be described by a single exponential decay of the *cis* state with a half time value of 4.0 h. The large mismatch between the slow *cis*-to-*trans*-reversed isomerization of azobenzene molecules and the fast density change of the polymer challenge the present theories. The fast relaxation of volume is confirmed by mechanical experiments at light-induced volume-expanded surface topographies. For instance, transient friction coefficient measurements demonstrated relaxation within 10 s after the actuating light source is switched off^18^. Also, a reference kinetic measurement of the height change as provided in Fig. 1b shows that both the appearance and the disappearance of additional volume evolves within 10 s.

### Single-wavelength versus multiple wavelength exposure

Taking notice of these observations, we propose a complementary mechanism in which the dynamics of the *trans*-to-*cis* and *cis*-to-*trans* isomerization process plays an important role other than populating *cis* isomer alone. This dynamics was proposed to be responsible for the lateral transport of azobenzene-modified side-chain polymers under the exposure with an interference pattern of a laser source^19^. Here we anticipate its importance in relatively densely crosslinked networks where the dynamics in the azobenzene isomerization enhances molecular voids in the polymer matrix and eventually towards a large volume increase on the macroscopic level. This proposed extension of the mechanism is supported by the observation that with a mercury lamp, which has a plurality of emission lines (promoting the backward reaction to the *trans* isomer), the decrease in density is a remarkable factor of 4 larger than exposed with 365 nm light alone at similar intensity. Together, with the current theory, we are now providing a more complete overview on the photomechanical effect in azobenzene-modified LCNs and their dynamics.

**Figure 2:**
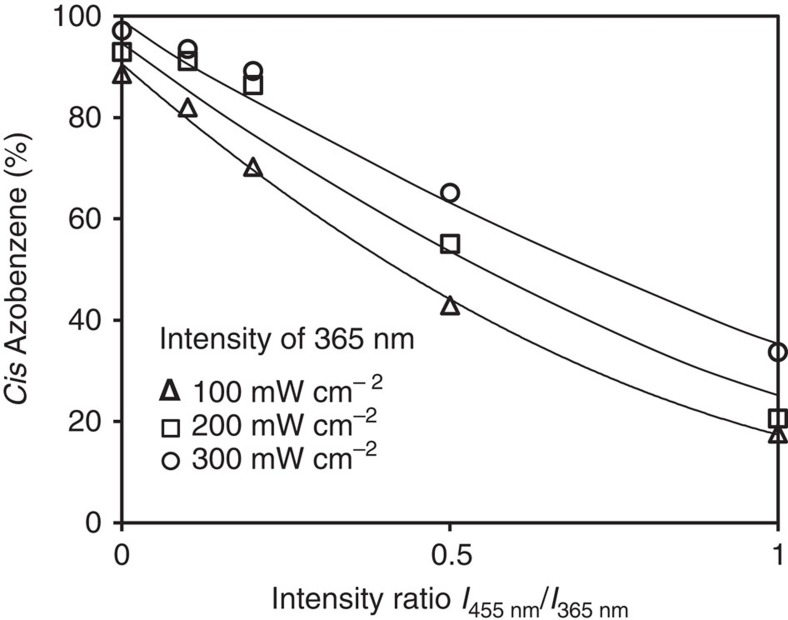
Photostationary state of azobenzene in the LCN. The equilibrium end conversion of *trans* azobenzene to its *cis* state under various light illumination conditions using a blend of 365 and 455 nm light, both generated simultaneously by an LED light source. The horizontal axis gives the intensity ratio of both wavelength.

For this study, we made a responsive LCN* coating by photopolymerization of a mixture of liquid crystal monomers in their chiral-nematic state on a glass substrate. Premature conversion of the azobenzene moiety is prevented by using blue light for polymerization. Further details are given in the Methods section and Supplementary Note 1. We studied the conversion of the azobenzene by ultraviolet illumination under various light and intensity conditions, as well as its relaxation at room temperature after switching off the light source. Figure 1a shows a sample that is actuated by an LED lamp emitting at 365 nm light with an intensity of 200 mW cm^−2^. The conversion of the covalently embedded azobenzene to the *cis* state exceeds 90 mol% at equilibrium. Relaxation in dark back to *trans* takes many hours with a half-life to 50% conversion of 4.0 h. The conversion under continuous exposure is defined as the photostationary state and is determined by the ratio between the forward and backward isomerization reactions. To affect this ratio, we added different intensities of 455 nm light to 365 nm light. The exposure routines are carried out at room temperature and using LEDs as near-monochromatic light sources. The half-intensity bandwidth of the LEDs used is 10 nm. The results are presented in Fig. 2 where the equilibrium photostationary state is plotted versus the ratio of the intensities of 455 and 365 nm. The percentage *cis* is calculated from the absorbance extremes as observed in Fig. 1a, assuming 1% in the dark and 96% conversion upon high-intensity 365-nm exposure. Reference measurements by nuclear magnetic resonance and ultraviolet–visible can be found in the Supplementary Figs 1–3 and Supplementary Note 2. For the measurement absorbance, we took a film of LCN* of exactly the same thickness but without azobenzene modification as baseline. The conversion in the photostationary state of 365 nm exposure alone is almost the same and close to full conversion. When adding 455 nm light, the conversion *trans* to *cis* becomes less, as anticipated as the back reaction is stimulated. At an 1:1 intensity ratio, the conversion is only around 30%. The experiments at the various absolute intensities gave somewhat different results with somewhat higher *cis* conversion at the higher intensities of both 365 and 455 nm. Apparently, the 365-nm intensity is dominating over the 455-nm, which might be understood from the larger absorption coefficient of the *trans* band. Of relevance for the experiments described below is that the intensity ratio of 455 nm/365 nm<0.2. The influence of the presence of 455 nm light on the photostationary state is still relatively small keeping them all at a *trans* conversion >80%.

When the azobenzene is activated by ultraviolet illumination, the LCN* coating undergoes a density decrease. To quantify this change, we use a photomask to measure the volume increase under illumination that manifests itself as protrusions formed on the coating surface. The width of the protrusions is chosen relatively large with respect to the height such that mechanical constraints of the volume increase in the centre is minimized as was previously checked by immersion density measurements^16^. By measuring the height of this protrusion, the density change *ϕ* is obtained and is calculated as 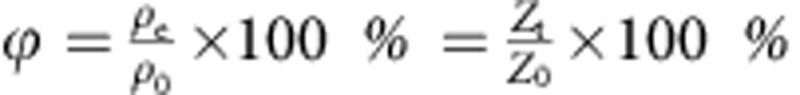, where *ρ*_e_ is the reduced density under ultraviolet irradiation, *ρ*_0_ is the initial density, which is measured by a density column at room temperature (RT) to be 1.217 g cm^−3^, *Z*_t_ is the height of the formed protrusions, *Z*_0_ is the initial coating thickness of 10 μm. The protrusions only form under ultraviolet illumination; they relax within 10 s as soon as the light is switched off before a quantitative measurement can be conducted. Therefore, we developed an indirect way of using polydimethyl siloxane (PDMS) (Sylgard 184-Dow Corning) replication to measure the protrusions. This procedure is illustrated in Supplementary Fig. 4 and Supplementary Note 3.

**Figure 3:**
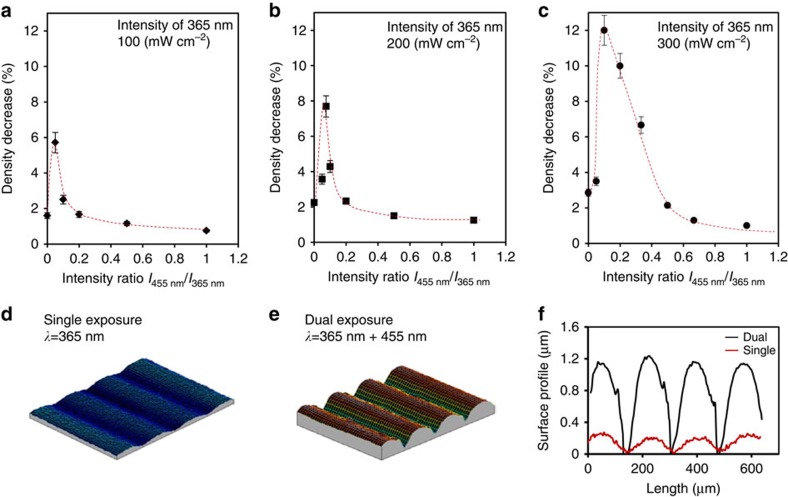
Density change and volume increase under various illumination conditions. (**a–c**) Decrease in density of LCN* under the different illumination conditions when 365 nm LED light is blended with 455 nm LED light. The error bars give the deviation within five separate measurements. (**d**,**e**) Interference microscopy measurements of the surface profile during (**d**) exposure to single 365 nm light, and (**e**) exposure to simultaneous 365 and 455 nm. (**f**) Corresponding surface profiles measured at a 10-μm film found during one-wavelength 365 nm exposure (red curve) and two-wavelength 365 nm +455 nm exposure (black curve).

The density change is measured under various intensities of near-monochromatic 365 nm light as generated by the ultraviolet–LED lamp. Under equilibrium illumination conditions, the density decreases to 1.193 g cm^−3^ at 100 mW cm^−2^ and 1.174 g cm^−3^ at 300 mW cm^−2^, corresponding to a density decrease of 2 and 3%, respectively (Fig. 3). And although Fig. 2 has shown that the intensity of the 365-nm ultraviolet–LED is of minor importance for the end conversion of the *trans* state to it equilibrium photostationary state, it is of influence on the volume increase and the corresponding density reduction.

In a following experiment, we focussed a blue LED lamp, emitting at 455 nm light, to the same spot as illuminated by the 365-nm LED. The results are plotted in Fig. 3a–c. Surprisingly, already at low intensity of added 455 nm light, we found a significant effect. The protrusion in the surfaces are significantly higher and as are the derived reductions of the density. Maximum density decreases are found when the 455-nm intensity is around 2.5, 5 and 10% of the 365 nm intensities at 100, 200 and 300 mW cm^−2^, respectively. Higher 365 nm intensity requires a higher 455-nm intensity to generate the maximum results. At the highest intensities, the density drop can be as high as 12%, which is a remarkable value. Also remarkable is that the effect drops fast at higher intensities of blue light. Obviously as soon as the *trans* conversion becomes too low, the effect diminishes.

An example is given in Fig. 3d–f, which compare the three-dimensional images of deformed surfaces, as induced by the density reduction, under single and dual-wavelength exposure. The sample in Fig. 3d is exposed to single 365 nm at 300 mW cm^−2^. Surface protrusions of 0.3 μm are observed on a 10-μm-thick coating, corresponding to a density decrease of 3%. The sample in Fig. 3e is exposed to the same intensity of 365 nm while 30 mW cm^−2^ of 455 nm light is included simultaneously. Now the protrusions increase to 1.2 μm for the same film thickness, corresponding to a density decrease of 12% at the top of the protrusion. Comparison of Fig. 2 with Fig. 3 leads to the conclusion that the conversion of the *trans* azobenzene to its photostationary state is not the only parameter for density reduction. Activation of the *cis*-to-*trans* cycle, as stimulated by the blue light, gives a significant extra stimulus to the density reduction and the corresponding protrusion formation.

We attribute the reduction of density in these highly ordered polymer networks to the formation of free volume as the order the molecular rods is reduced. Free volume formation in azobenzene side-chain polymers by photoactuation is published earlier by Barrett and co-workers^20^. They reported even a volume increase of 17% at room temperature, measurements that were supported by neutron reflectometry. In our case, the azobenzene content was much lower, 2% of the acrylate monomers units contain an azobenzene moiety versus 100% in the Barrett paper. Moreover, we perform our experiments at crosslinked polymer networks rather than with linear polymers. Free volume effects in free-standing bending films made of liquid crystal polymer networks were reported by the Ikeda and co-workers^21^.

We propose that the cycle dynamics of the azobenzene with a repetitive forward and backward isomerization reaction plays an important role in the process of free volume creation. This dynamics is further enhanced by exposing the azobenzene simultaneously in its *trans* and *cis* absorption band, bringing the photostationary ratio between forward and backward reaction out of its chemical equilibrium. As soon as the exposure stops, the azobenzene still remains largely in its *cis* state but the network, being far out of its viscoelastic equilibrium, directly relaxes back to its flat high-density state. One might debate whether the volume increase is the result of a purely thermal effect as caused by heating of the sample upon absorption of light by the azobenzene moieties. Thermal volume expansion coefficients of the chiral-nematic networks were reported earlier^16^ to be 285 p.p.m. below and 552 p.p.m. above the *T*_g_ of 60 °C. If we base the volume expansion purely on heating, we need a temperature rise of 70 °C to explain 3% expansion and 236 °C for the 12% expansion as reported in Fig. 3c while in experiments we measured a temperature increase from 20 to 28 °C by infrared camera. We also performed control experiments by exchanging the azobenzene by a photomechanically inactive dye (Supplementary Fig. 5 and Supplementary Note 4) and could measure an expansion of <1%.

### Side chain compared with crosslinking azobenzene

**Figure 4:**
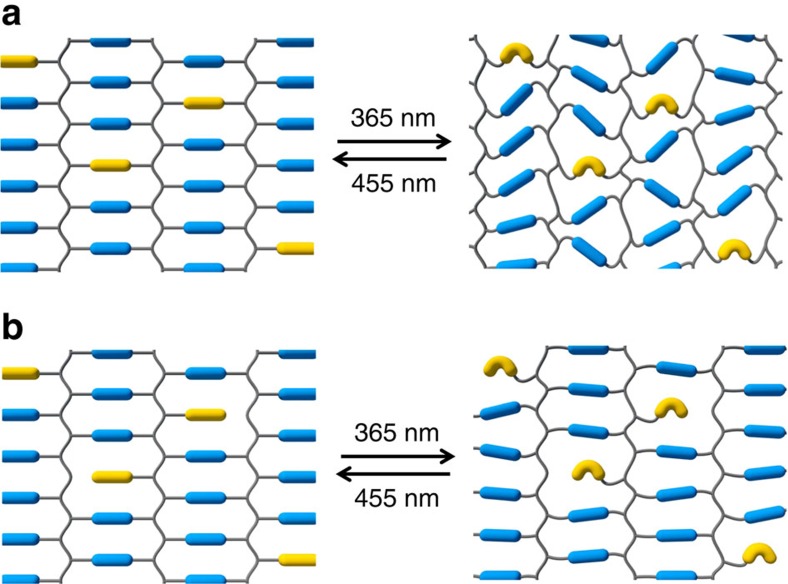
Schematic view of oscillatory LCN deformation. Volume increase induced by copolymerized di-acrylate azobenzene (**a**) and mono-acrylate azobenzene (**b**).

The gained insights in the generation of molecular voids in LCNs raises another fundamental question whether they originate from the dynamic motion of the entire network, as the azobenzene deforms the main chain of the network during its dynamic isomerization, or whether the geometrical changes of azobenzene molecules alone are capable to create space, that is, the space it needs for its isomerization to occur? To answer this question, we compared the di-acrylate azobenzene with a mono-acrylate azobenzene. The di-acrylate connects to the polymer network at its both ends. The oscillation of the *trans–cis* conversion drives the entire network into a simultaneous distortion (Fig. 4a) while the mono-acrylate azobenzene is only linked at one end to the network. Consequently, the conformational changes of the azobenzene are decoupled from the network during its isomerization process. Therefore, it would only create its volumetric space for its reaction while exerting less force on the network as a total as illustrated in Fig. 4b. The results show that the local volume increase induced by mono-acrylate azobenzene is <2% compared with the 12% volume increase under the same conditions when using the azobenzene di-acrylate. These results are conflicting with the observations by Ikeda and co-workers who described a system in which free volume formation is higher for the non-cross-linking azobenzene monomers^21^. However in their system free-volume formation and order reduction work in opposite direction. Here we showed surface deformation in a planar-oriented chiral-nematic network where the reduction of order parameter and free volume expansion both work in concert towards an out of plane deformation perpendicular to the film surface.

The cycle time of the measured deformation is in the order of 0.1 Hz. An estimate of the cycle rate of the stimulated *trans–cis* conversion based on the number of absorbed photons per unit of volume^22^ is of the order of 1 kHz. These four orders of magnitude difference further support the assumption that the response rate, as for instance, demonstrated in Fig. 1b is dominated by the viscoelastic deformation of the polymer network rather than by the photochemistry. Recently published athermal photofluidization phenomena in azobenzene polymer glasses might further support the possibility of deformation of the glassy network at room temperature^22^.

### Fluorescent dye-enhanced actuation

**Figure 5:**
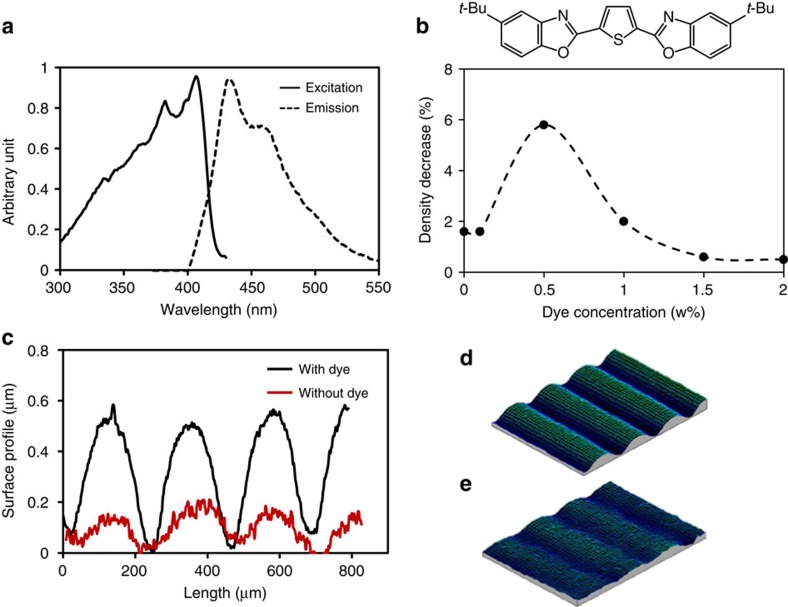
Comparison of LCN* actuation with and without fluorescent dye. (**a**) Excitation/emission of the fluorescent dye. (**b**) Chemical structure of the fluorescent dye and comparison of the density decrease at various dye concentrations. (**c**) Interference microscopy measurement of the surface deformation of a 10-μm LCN* coating with dye (black line) and without dye (red line), and (**d,e**) the corresponding three-dimensional surface profiles.

To further support our hypothesis and to provide a simple experimental routine that allows a single LED exposure other than focussing two LEDs on the sample, we convert a small fraction of 365 nm light to 455 nm by adding a low concentration of fluorescent dye. This dye absorbs 365 nm light and emits light around 440 nm; Fig. 5a,b and Supplementary Note 1. The experiment was performed under 365 nm illumination at 100 mW cm^−2^. The results shown in Fig. 5c–e demonstrate a large effect in comparison with the reference sample without the fluorescent dye. The optimum dye concentration is around 0.5 w%. Under this condition, the calculated density decrease of the sample with dye corresponds to the experiment where an intensity of 365 nm at 100 mW cm^−2^ was combined with optimum additional 455 nm at 2.5 mW cm^−2^.

## Discussion

We have demonstrated that dual-wavelength exposure creates a large surface deformation of an azobenzene-activated liquid crystal polymer coating. Instead of involving solely 365 nm light to excite azobenzene, we include a small fraction of 455 nm light. This wavelength corresponds to the absorption band of the *cis* isomer. It is postulated that this promotes the back conversion to the *trans* isomer and the related oscillatory dynamics of the isomerization reaction. Remarkable high surface protrusions are formed under these conditions up to 12% of the initial film thickness. This should be compared with the 3% when exposed by single wavelength of 365 nm light. The surface protrusions originate from an overall density decrease by the formation of molecular voids (free volume). This is further boosted by the accelerated dynamics of the azobenzene *trans–cis* and *cis–trans* isomerization processes. We have further investigated that the origin of the free volume formation is due to the dynamic motion of the entire polymer networks other than the conformational changes of azobenzene alone. As soon as light is switched off, the oscillatory dynamics ceases and the free volume relaxes fast because of its thermodynamic instability. Despite the fast mechanical relaxation on the timescale of seconds, the chemical relaxation of the *cis* azobenzene to its *trans* state is lagging behind by hours.

We anticipate that these results provide a better insight in the photoactivation of azobenzene-modified liquid crystal polymer actuators in general. It demonstrates how by affecting the molecular dynamics of single molecules, only present in a relatively low concentration, affects the macroscopic changes of the whole polymer. As an example, we developed an enhancement of the single-wavelength actuation deformation by the addition of a small amount of fluorescent dye to the azobenzene-driven mixture.

## Methods

### Materials

**Figure 6:**
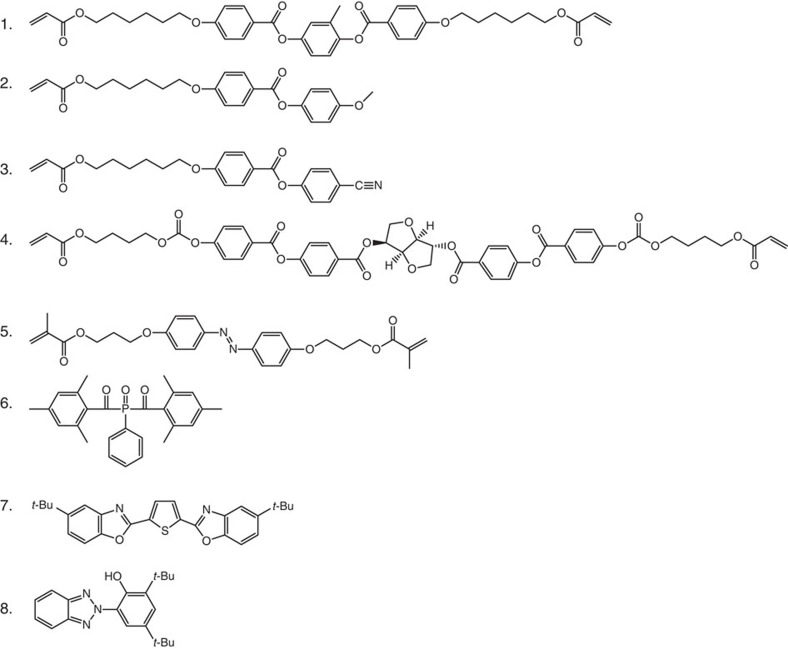
Applied materials. Liquid crystal monomers (**1-3**), polymerizable chiral dopant (**4**), polymerizable azobenzene (**5**), photoinitiator (**6**), fluorescent dye (**7**), inert ultraviolet absorber (**8**).

The mixture is composed of a blend of liquid crystal acrylates and some additives as shown in Fig. 6. Details of the composition are presented in the Supplementary Note 1. Monomers **1–3** exhibit a nematic phase in which the molecules tend to align with their long axes along a common director. In this mixture, the mono- and acrylates are carefully balanced to optimize the properties at both monomeric and polymeric states. Important parameters for the monomeric state are low crystalline melting temperature, a wide nematic phase to provide a broad processing window enabling film formation and good solubility in organic solvents. Di-acrylate **1** is a crosslinker that is used to balance the mechanical properties of the polymer. Its concentration is adjusted such that the glass transition temperature of the ultimate polymer network is well above room temperature. Monomers **2** and **3** enhance alignment properties and optimize the elastic properties of the LCN* on light-induced deformation. Chiral component **4** is added to induce the desired chiral-nematic phase where the average molecular orientation describes a helix. The helical pitch can be accurately adjusted by the concentration. Here 3.4 w% gave a helical pitch to be 652 nm. Monomer **5** has the azobenzene unit that provides the photomechanical response to the LCN*. Previously, we have found that a small amount of 2 w% is sufficient to produce significant effect^6^. Using low concentration of azobenzene not only reduces heating by light absorption but also limits the attenuation of light intensity along the coating thickness during actuation. Photoinitiator **6** is chosen as it can be activated by wavelengths >400 nm, preventing premature conversion of azobenzene compound during the photopolymerization process. Compound **7** is a fluorescent dye that is used to convert 365 nm light to 455 nm light. Compound **8** is a photomechanical inert dye that is used to confirm whether the large density decrease is originated from the photomechanical effect rather than the heat (Supplementary Fig. 5 and Supplementary Note 4). A mixture of these materials is applied between two glass substrates provided with rubbed polyimide. The glass plates were briefly sheared along the rubbing direction to promote planar alignment of the chiral-nematic liquid crystal mixture. After photopolymerization by light exposure at a wavelength >400 nm, the sample was subjected to a 30-min postbake at 120 °C to ensure full conversion of the acrylate groups as confirmed by Fourier transform infrared spectroscopy (FTIR). After cooling down to room temperature, the obtained polymer coating is in its glassy state.

### Process and characterization

The liquid crystal (LC) mixture was filled between two glass plates provided with the alignment layer by capillary suction. The sample was cured at 36 °C by ultraviolet exposure for 5 min with an intensity of 400 mW cm^−2^ using a mercury lamp (Exfo Omnicure S2000) equipped with a cutoff filter transmitting light >400 nm (Newport FSQ-GG400 filter). The samples were postbaked at 120 °C under N_2_ to ensure full conversion of the acrylate monomers. The thickness of the final polymer coating is 5 μm. The cholesteric film was checked by polarized microscopy (Leica). A LED lamp (Thorlab, M365L2 and M455L3) is used to provide monochromatic light. The surface topography is measured using an interference microscopy (Fogal Nanotech Zoomsurf).

## Additional information

**How to cite this article**: Liu, D. *et al*. New insights into photoactivated volume generation boost surface morphing in liquid crystal coatings. *Nat. Commun.* 6:8334 doi: 10.1038/ncomms9334 (2015).

## Supplementary Material

Supplementary Information Supplementary Figures 1-5 and Supplementary Notes 1-4
