# Supplementary material for: New insights into photoactivated volume generation boost surface morphing in liquid crystal coatings
Source: Nat Commun. 2015 Sep 21;6:8334. doi: 10.1038/ncomms9334 (PMC4595720; doi:10.1038/ncomms9334)
Supplement: Supplementary Information — Supplementary Figures 1-5 and Supplementary Notes 1-4 [file ncomms9334-s1.pdf]

Supplementary Figures

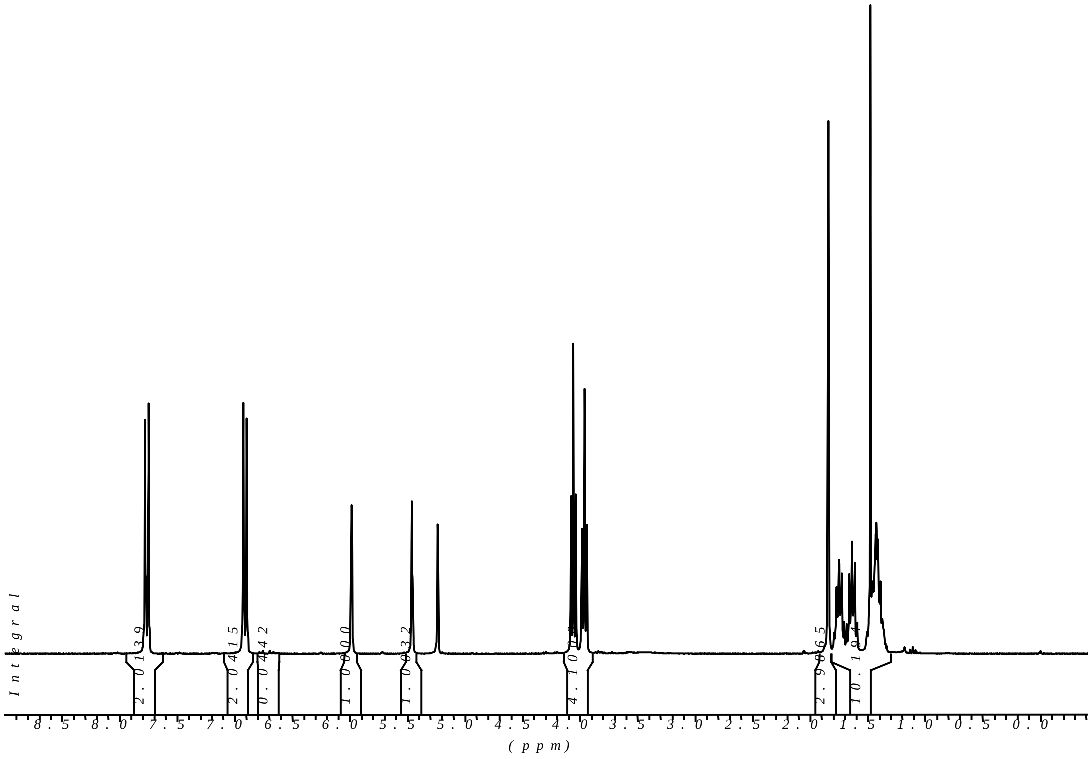

Supplementary Figure 1. Monomer **5** in CD_2Cl_2, 99% *trans*-isomer

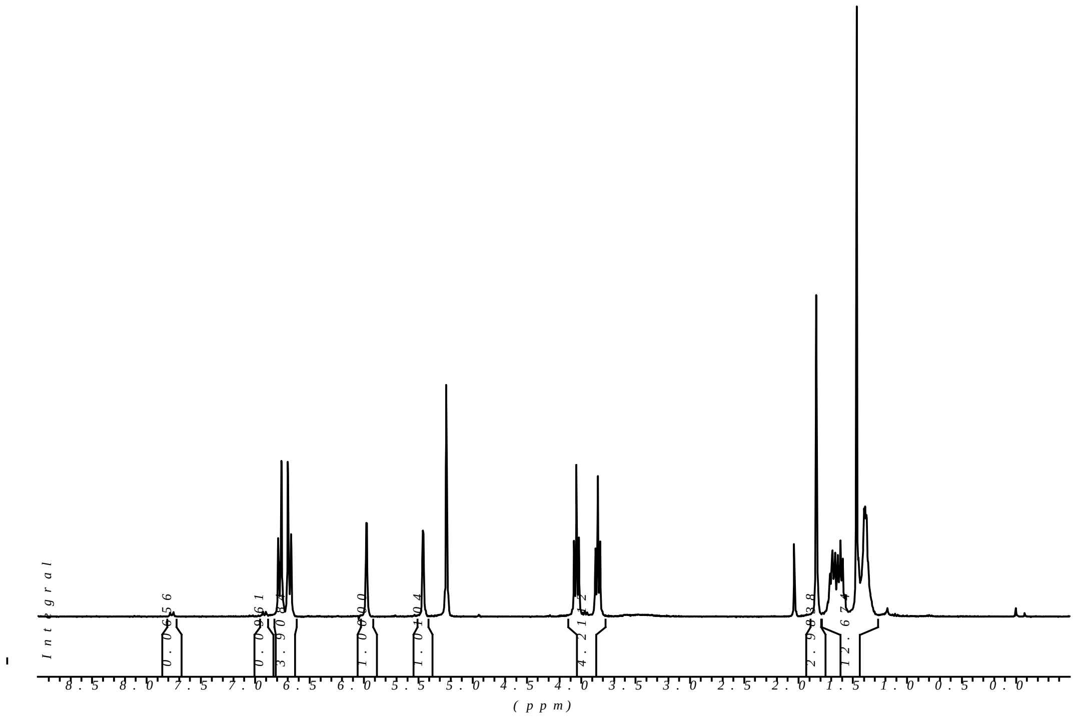

Supplementary Figure 2. Monomer **5** in CD_2Cl_2, 99% *cis*-isomer

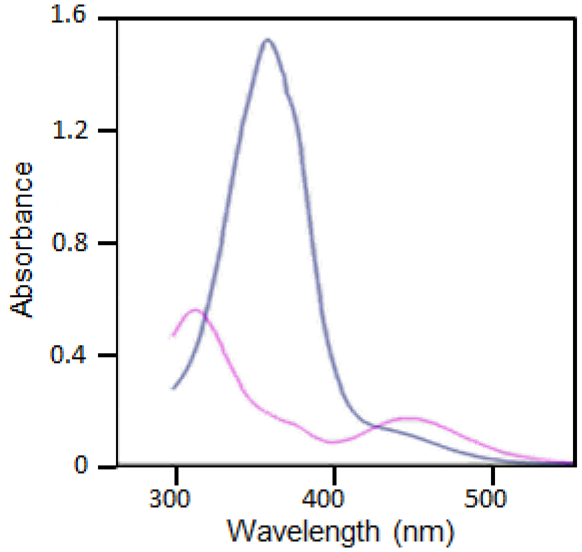

Supplementary Figure 3. Absorption spectra of the irradiated (365 nm red curve) and un-irradiated (blue curve) sample of $5.12\cdot10^{-5}$ M **5** in CH_2Cl_2.

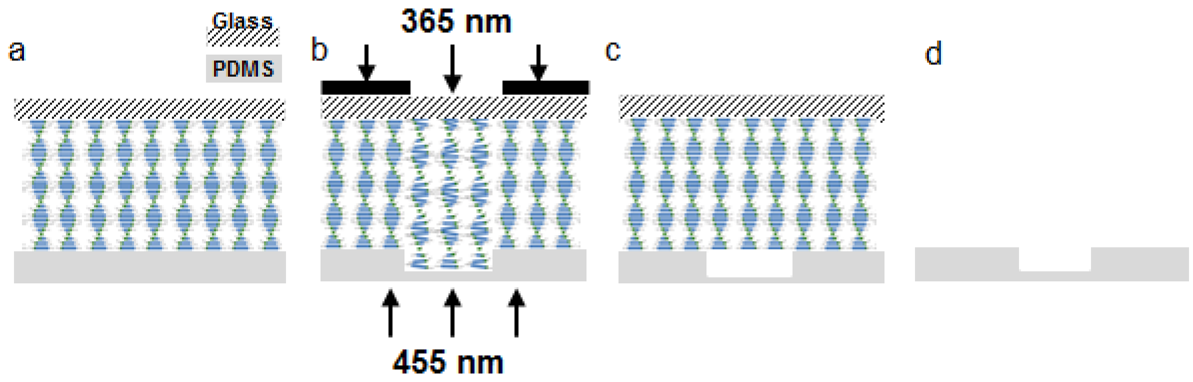

Supplementary Figure 4. Schematic representation of the PDMS procedure to record local volume increase of the LCNs coating. **a,** Liquid PDMS is casting on the flat LCNs coating. **b,** Exposure through photomask and cure PMDS. **c,** Solidified PDMS captures the increased volume of the coating when UV stopped and **d,** separate PDMS replication from the coating.

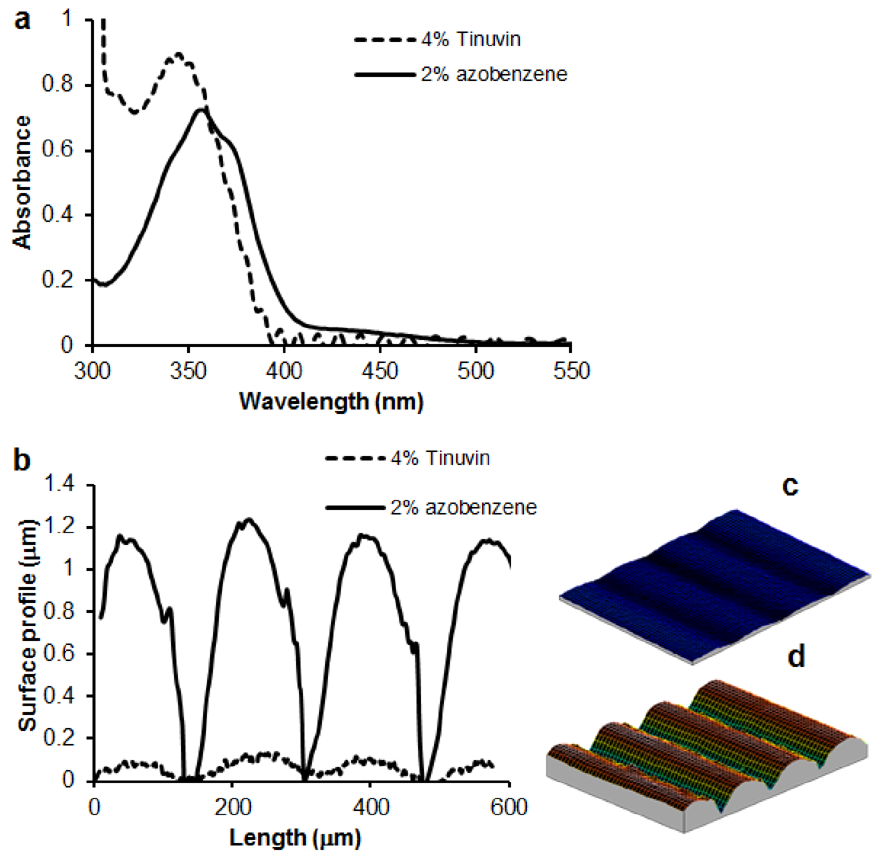

Supplementary Figure 5. Comparison between thermal effect and photomechanical effect. **a,** Absorbance of azobenzene and the Tinuvin. Induced density differences by thermal effect (**b** (dashed line), **c**) and photo-mechanical effect (**b** (solid line), **d**).

Supplementary Notes

Supplementary Note 1:

Materials and Methods

Materials. Scheme 1 shows the components for our reactive mixtures. Liquid crystal monomers **1** to **3** were obtained from Merck UK. Fluorinated monomer **4** was obtained from Sigma Aldrich. Photoresponsive monomer **5** was custom-synthesized by Syncom (Groningen, The Netherlands). Photoinitiator **6** was purchased from Ciba Specialty Chemicals. Typically, thin films were fabricated from a mixture containing 20 w% **1**, 42.7 w% **2** and 31.4 w% **3**, 3.4 w% **4**, 2 w% **5**, 1 w% **6**. The constituents were mixed by dissolving in dichloromethane. DSC results show that this mixture has a melting temperature of 40 ℃ above which it becomes nematic. Above 60℃ the mixture is isotropic. Fluorescent dye **7** 2,5-bis (5-tert-butyl-benzoxazol-2-yl) thiophene (Sigma Aldrich) was later added to the mixture. To achieve 6% density decrease under the 365 nm exposure, 0.5 w% was added.

Sample preparation. Glass substrates are cleaned by a 5 minutes dip in acetone under stirring, 5 minutes in propanol-2 under stirring, flushed with demi water followed by drying with a nitrogen flow. A1051 (Sunever, Nissan Chemical, Japan) was used to obtain planar alignment of the liquid crystal monomer mixture. It was spin coated on cleaned glass followed by baking. Gentle manual rubbing on a velvet cloth gave the substrate the desired alignment.

The LC mixture was filled between two glass plates provided with the alignment layer by capillary suction. The sample was cured at 36°C by UV exposure for 5 minutes with an intensity of 400 mW.cm^{-2} using a mercury lamp (Exfo Omnicure S2000) equipped with a cut-off filter transmitting light > 400 nm (Newport FSQ-GG400 filter). The samples were post-baked at 120℃ under N_2 to ensure full conversion of the acrylate monomers. The thickness of the final polymer coating is 5 μm.

Sample characterization. The cholesteric film was checked by polarized microscopy (Leica). A LED lamp (Thorlab, M365L2 and M455L3) is used to provide monochromatic light. The surface topography is measured using an interference microscopy (Fogal Nanotech Zoomsurf).

Supplementary Note 2:

Conversion of azobenzene monomer 5

A point of possible discussion is the purity of 100% *trans* azobenzene monomer 5 before exposure and the conversion in the photostationary state. From NMR spectra of the monomer dissolved in CD_2CL_2 we concluded the monomer **5** as synthesized is for 99% in its *trans* state (Supplementary Figure 2).

By exposing with UV light we could convert this monomer for 96% to its *cis* state (Supplementary Figure 3). Simultaneously we measured the conversion of the azobenzene monomer in dichloromethane by UV VIS before and during UV exposure with 365 nm light (Supplementary Figure 3) from which derive the conversion data for our solid state experiments assuming the extinction of the azobenzene moiety remains the same.

Supplementary Note 3:

PDMS replication process

The procedure for the replication of the activated surface is schematically shown in Supplementary Figure1. The liquid PDMS (Sylgard 184-Dow Corning) precursor was poured on the surface of the polymer coating. Curing of the PDMS takes place while the polymer coating is mask exposed to replicate the forming surface structure. The curing of PDMS takes 48 hours at room temperature. After solidification PDMS was peeled from the coating surface and can then be measured by interference microscopy which gives the indication of density decrease. Although PDMS has lower surface energy than the air, the effect of which can be ignored considering aspect ratio (height/width) of the formed structure is very small <0.1.

Supplementary Note 4:

Thermal effect vs. photo-mechanical effect

When the samples are exposed to UV light some heating cannot be excluded. To estimate the influence of heating, we exchanged the photomechanical azobenzene monomer by compound **8** (Tinuvin-BASF), which has the same absorbance at the wavelength of 365 nm. Supplementary Figure 5(a) compares the absorption spectra in the azobenzene sample and the reference sample with Tinuvin. Both sample are subjected to the dual wavelength exposure. Intensities of 365 nm at 300 $mW.cm^{-2}$ and 455 nm at 30 $mW.cm^{-2}$. In Supplementary Figure 5(b-c) the surface profiles are given of Tinuvin modified LCN*(b (dashed line), c) and the azobenzene containing LCN* (b (solid line), d). As can be seen, in case of Tinuvin modified coating, the density decreases 1.1% compared with azobenzene containing film where 12% of density decrease is found. We also know from previous work that below T_g the thermal expansion coefficient of this polymer is 285 ppm. A volume increase of 1.1% implies that our sample is heated up less than 20°C. This made us conclude that thermal effects alone do not play a major role. However it cannot be excluded that in the case of azobenzene some heating of the sample might enhance the photomechanical effect.
